# Early Prediction of Sepsis Based on Machine Learning Algorithm

**DOI:** 10.1155/2021/6522633

**Published:** 2021-10-12

**Authors:** Xin Zhao, Wenqian Shen, Guanjun Wang

**Affiliations:** School of Mathematics, Southeast University, Nanjing 211189, China

## Abstract

Sepsis is an organ failure disease caused by an infection resulting in extremely high mortality. Machine learning algorithms XGBoost and LightGBM are applied to construct two processing methods: mean processing method and feature generation method, aiming to predict early sepsis 6 hours in advance. The feature generation methods are constructed by combining different features, including statistical strength features, window features, and medical features. Miceforest multiple interpolation method is applied to tackle large missing data problems. Results show that the feature generation method outperforms the mean processing method. XGBoost and LightGBM algorithms are both excellent in prediction performance (AUC: 0.910∼0.979), among which LightGBM boasts a faster running speed and is stronger in generalization ability especially on multidimensional data, with AUC reaching 0.979 in the feature generation method. PTT, WBC, and platelets are the key risk factors to predict early sepsis.

## 1. Introduction

According to the sepsis-3 criteria, sepsis refers to an out-of-control body reaction caused by infection, leading to life-threatening organ failure. Patients suffering from sepsis face a terribly high risk of death. A survey [1] shows that the number of people who die from sepsis worldwide is higher than previously thought. In poor areas, the vast majority of the dead are children. In 2017, there were 48.9 million sepsis cases worldwide, of which 11 million died of infection, and the mortality rate was as high as 20%. At present, it is a pity that few antiseptic treatment programs have been clinically proven to be effective.

In intensive care, early recognition of the sepsis risk is essential to control the disease, because the treatment of sepsis is highly time-sensitive. According to the International Medical Guidelines, it is recommended to start early fluid resuscitation within the first 3 hours to stabilize tissue hypoperfusion induced by sepsis, and it is recommended to start intravenous antibacterial agents as early as possible, especially within 1 hour after sepsis. Actually, every hour of delay in treatment will increase the mortality rate by about 4–8%. In order to grasp the “golden period” of controlling disease progression, rule-based sepsis scoring systems are usually used in clinical settings, including systemic inflammatory response syndrome (SIRS) criteria, sequential organ failure assessment (SOFA) scores, and modified early warning score (MEWS), to alert the possible occurrence of sepsis. By providing timely interventions, these early warning scores can help with early warning programs or specific prehospital treatment with high sensitivity. However, these criteria are poor in specificity. For example, the physiological indicators of viral influenza can often cause false alarms.

Artificial intelligence technology emerges as an effective method in medical assistance including early sepsis diagnosis. Through the integration of electronic medical records, medical imaging, pathophysiology, and other data, these methods have been developed to analyze and predict the health of the human body and obtain accurate prescription information to help clinicians make quick and effective judgments.

## 2. Literature Review

A diagnosis system based on artificial intelligence (AI) is shown to be effective in many medical fields. In the area of diagnosis, prognosis, and treatment of sepsis, machine learning algorithms used include supervised learning and reinforcement learning [2–5]. For example, Beck et al. [6] develop the C-Path (Computational Pathologist) system to automatically diagnose breast cancer and predict whether patients will survive or not by examining breast tissue imaging.

The main two challenges in the current research include the use of different physiological indicators and modeling efficient machine learning algorithms for the diagnosis, prognosis, and treatment of sepsis. Similarly, in order to predict sepsis in advance, it is also crucial to choose appropriate variables and design valuable algorithms in the clinical setting.

The input variables of the model are physiological indicators and the output variable is whether the patient would suffer from sepsis several hours later. Specifically, the input variables generally include vital signs like heart rate, oxygen saturation, and body temperature; biomarkers like procalcitonin and interleukin-6; laboratory values like bicarbonate and creatinine; and demographic variables like sex and age. In most cases, the variables include lots of missing values, such as that in MIMIC III (Intensive Care Medical Information Market Database), which has been used in many studies. Among most researches, variables with lots of missing values are excluded from predictors, so valuable information may be lost as a result. Several studies use imputation and mean filling methods to fill in missing values, but this may also lead to selection bias or mixtures of confounding factors. The data preprocessing method needs to be considered according to the characteristics of different data sets.

Common ways to deal with missing values are missForest [7], KNNimpute [8, 9], and so on. Other ways are also proposed. For instance, Desautels et al. [10] proposed the InSight algorithm by using easy-to-monitor patient vital signs data and an integrated tree boost algorithm to train the model so as to simplify the types of input variables as much as possible. The final simplified input variables include vital signs (systolic blood pressure, pulse pressure, heart rate, respiratory rate, temperature, and peripheral capillary oxygen saturation (SpO_2_)), patient age, and Glasgow coma score (GCS). Its AUC indicating discriminative power between infected and noninfected patients reaches 0.880. Taneja et al. [11] make a detailed comparison of input variables such as vital signs and biomarkers and predict sepsis risks 4 hours in advance. The vital signs and biomarkers are separately used as input variables to train the model to obtain the AUC score, and then, they are both used as input variables to train the model to compare the effects. The final feature importance is listed in order as vital biomarkers and vital signs.

The machine learning algorithms generally include support vector machines, gradient boosting trees, random forests, Lasso regression, and neural networks. Among them, support vector machines and gradient boosting trees have shown good performance. The model with better prediction ability will be further tested and improved for clinical service so that clinicians can make better decisions in sepsis early diagnosis. Taneja et al. [11] compared the predictive abilities of five machine learning models, including logistic regression, support vector machines, random forests, Adaboost, and Naive Bayes. Among them, the support vector machine algorithm and Adaboost algorithm have the highest AUC scores. The other models in use also include deep learning methods and biological methods. For example, Scherpf et al. [12] used a recurrent neural network (RNN) to conduct experiments on the sepsis data set provided by the MIMIC III platform. Nemati et al. [13] used a proportional hazard model to predict sepsis several hours in advance. Lin et al. [14] used the convolutional LSTM model, the random forest model selected by Lamping et al. [15], and the Gaussian process-based RNN model used by Hariharan [16].

The above studies have shown good performance in the field of sepsis prediction. However, the amount of data used in these researches are shrunk, as most of the missing values are processed by direct deletion or forward filling, and the explanatory ability of the model is also limited. It is challenging to transfer these methods into clinical practice for the following detailed reasons. (1) A unified data set is lacked. Researchers use data from different patient groups, for example, the MIMIC public database or other independent hospital data sources. The clinical variables they select to generate models differ and the scale of data differs a lot as well. (2) The premise and indicators of prediction settings vary, such as clinical standards for sepsis, observation windows, and evaluation indicators.

Above all, it is still not possible to do full validations for sepsis prediction in different groups with current machine learning methods and evaluate their generalizability. In addition, many of the machine learning models are complex and hard to be explained. Clinicians lack tools to interpret this “black box” model in clinical practice. This study is committed to digging out the most effective information from large-scale data. In terms of the interpretability ability, a metric called SHAP value is used in this study which can help models break the “black box” barriers and have good interpretability.

Specifically, this research develops machine learning models with good generalization ability and clinical interpretability by generating two data preprocessing methods based on XGBoost and LightGBM algorithms, which can be used to predict early sepsis 6 hours in advance, to assist clinicians in early diagnosis, intervention, and treatment. (1) In the mean processing method, it is explored whether or not the model predictive ability will be improved by extracting mean vectors. After dividing the early warning period into 2 hours or 3 hours window, it is discussed about the relationship between the extent of category imbalance and the model's predictive ability. (2) In the feature generating model, the prediction performance of raw variables trained in different models are compared with those extra with different types of newly generated features in the relationship between model performance and model complexity.

The rest of the research is arranged as follows. In Section 3, materials and methods are given. The data used for prediction are introduced, followed by the two data processing methods and the prediction process.  Section 4 reports the results of predictive analysis and explores the complexity of data preprocessing, as well as the number and types of new features generated which affected the model's prediction ability.  Section 5 gives the conclusion and future work.

## 3. Materials and Methods

The original data are from the physiological ICU database from three independent hospital systems [17, 18], making a total of 22336 patients (1714 sepsis patients). The frequency of the data shown is one hour, making a total of 790,125 observations. The data set has 40 indicators, including 8 vital signs, 26 laboratory values, and 6 demographic indicators as shown in Table 1. Instead of one hour, most of the laboratory values are measured per 12 hours or per day, resulting in about 90% missing values due to the difference in the data collecting frequency. Besides, the data collected in this paper is based on the latest definition of diagnosis of sepsis 3.0.

It can be seen from Table 1 that the basic information of the demographic indicators is fairly complete, and most vital sign indicators are frequently measured, with a relatively low proportion of missing values. On the contrary, laboratory variables, involving biomarkers, have a long-time gap of collection intervals, and most of the values are missing. If missing values are deleted directly, a lot of information will be lost. That is helpless at sepsis prediction, and this study uses the imputation method to fill missing data instead of deleting the variables directly.

There is also a group imbalance problem involved. Among the 22336 patients, there are only 1714 patients suffering from sepsis, and in the 790215 observations of original data, there are only 17,135 observations with sepsis label as 1, and the rest as 0. The ratio of 0-1 categories is 45 : 1 as shown in Table 2. The response variable has an obvious 0-1 distribution imbalance, which intensifies the difficulty of predictive modeling. If it is forcibly modeled, the algorithm will return a learner that always predicts a new sample into category 0. This study will preprocess the original data to deal with the problem of data imbalance and missing values, by applying the mean processing method and the feature generation method.

### 3.1. Mean Processing Method

The observation labels of patients include the state of no illness, 6 hours before the illness, and the state of illness, which are, respectively, called the safe period, the early warning period, and the sick period. The values of these three states are set as 0, 1, and 1, respectively. The reason that the sepsis label in the early warning period is also marked as 1 is that the goal of the study is to predict the onset 6 hours in advance, so the warning period is also marked as 1. Due to the problem of large missing values, the data of patients who suffer from sepsis are transferred into three observations labeled with 0, 1, and 1. Each corresponding input variable is also averaged into three observations according to the range of label values. Such processing method could also help fix the problem: the lack of special biomarkers caused by a too long time interval. At the same time, for patients who do not suffer from sepsis, it is believed that values of their biological indicators were basically within the safe range and thus belonged to the safe period. Therefore, the data is averaged into one observation for each variable of each patient who does not suffer from sepsis.

After data processing, 23711 physiological data are finally formed. The 0-1 observation number of the sepsis label is 20133 : 3578, and the ratio is about 5.6 : 1. The category imbalance has been significantly improved.

#### 3.1.1. Feature Selection

In the mean processing method, 25 variables were determined to participate in the training model, including (a) vital signs indicators (HR, O_2_Sat, Temp, SBP, MAP, DBP, Resp), (b) laboratory variables (HCO_3_, pH, PaCO_2_, AST, BUN, AlkalinePhos, Chloride, Creatinine, Lactate, Magnesium, Potassium, Bilirubin_total, PTT, WBC, Fibrinogen, Platelets), and (c) demographic indicators (Age, Gender).

Variables with more than 98% missing proportions were removed. HospAdmTime (the time between hospitalization and ICU) and ICULOS (ICU hospitalization time) in the demographic indicators are deleted. HospAdmTime presents different numerical levels according to the condition of different patients and may be related to the longer incubation period of sepsis. This study is more interested in finding rules to predict early sepsis from the changes in specific physiological data, and they are eliminated to avoid being interfered. Patients with sepsis in the entered data face a high mortality rate. They often require long-time treatment in the ICU, and the ICULOS value is generally too high. On the contrary, patients without sepsis are generally treated in the ICU for only a short time and then transferred out of the ICU after the condition is improved, thus with low ICULOS value. The difference in ICULOS value is due to the difference in the nature of the illness condition, which is contrary to the causal sequence of early sepsis predicted from physiological data, so the variable ICULOS is deleted.

#### 3.1.2. Imputation of Missing Data

Missing data has a greater impact on data analysis, which is mainly manifested in two aspects: the weakening of data statistics and biased estimation. Kim and Curry [19] found that when 2% of the data is missing, deleting the missing value will bring about an 18.3% lack of information. Quinten [20] has shown that 10%∼35% of missing data will bring about 35%∼98% lack of information. Therefore, the direct deletion of missing values is only suitable for data sets with a low percentage of missing values and is generally not preferred.

The imputation method is divided into the single imputation method and the multiple imputation method. Single imputation is the simplest method, replacing missing values with a single value, without any estimation of the uncertainty of imputation. It is more accurate to use a single imputation method to fill a data set when the percentage of missing data is low. Multiple imputation is to consider the uncertainty of imputation by running a single imputation multiple times, so it can provide a more accurate estimate of missing data. These methods estimate incomplete data sets many times, by using standard analysis methods to analyze the estimated data sets. The results obtained from the analysis are finally aggregated into a result with less deviation. The multiple imputation method is more suitable for data sets with a high percentage of missing data.

Therefore, this article will use the multiple imputation method Miceforest [21] to impute the missing data. It is based on the multiple imputation of the chain equation of random forest, using the process of predictive mean matching to select the value to be estimated. The imputation method boasts a fast speed, with high memory utilization, and can output diagnostic maps and fill in missing data with high accuracy. Using the Python language as the tool and the Miceforest as the basis, the Multiple Imputed Kernel function is used to perform multiple imputation according to the missing percentage of various indicators.

**Figure 1:**
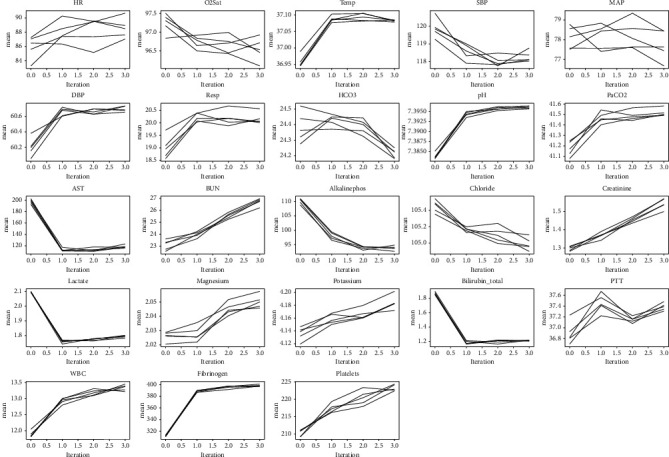
The result of convergence in the process of variable interpolation.

In Figure 1, the imputed mean line chart is plotted to see if the mean has converged. It shows that most of the 23 variables tend to converge in the mean after few iterations. This also confirms that the lack of patient physiological data in the data set is not completely random but is based on the existence of a regular lack of time interval.

**Figure 2:**
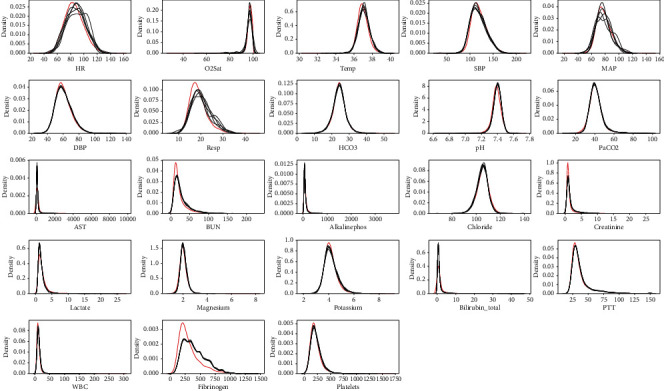
The data distribution after interpolation and the original data distribution.

After processing, the distribution of the original data and the data set after imputation is shown in Figure 2. Among them, the red line is the original data, and the black line is the imputed (estimated) value of each data set. It can be seen from the figure that the distribution of the 23 variable imputation data is similar to the original data, and it is intuitively shown that the fitting effect is good.

#### 3.1.3. Machine Learning Methods to Predict Early Sepsis

Two integrated tree algorithms are considered, XGBoost and LightGBM. The detailed information about these methods can be referred to [22–27]. The metrics precision, recall, F1-score, Kappa coefficient, and Matthew's coefficient are used to evaluate the prediction performance of the algorithm. The feature importance score and SHAP value are chosen to explain the model.

For the feature importance score, both XGBoost and LightGBM algorithms can output feature importance, which can intuitively reflect the importance of each feature in the data set through the score. The calculation formula of feature importance is shown below, and the importance of feature *x*_*j*_ in the entire model is

(1) $$\begin{array}{c} \hat{I_{j}^{2}}=\frac{1}{M}\sum_{m=1}^{M}\hat{I_{j}^{2}}\left(T_{m}\right). \end{array}$$

Among them, *M* is the number of trees in the model, and *T*_*m*_ represents the *m*^th^ tree.

The feature importance of feature *x*_*j*_ on a single tree is

(2) $$\begin{array}{c} \hat{I_{j}^{2}}\left(T\right)=\sum_{t=1}^{L-1}\hat{I_{t}^{2}}I\left(v_{t}=j\right). \end{array}$$

Among them, L-1 is the number of nonleaf nodes of the tree, *v*_*t*_ represents the feature selected when the internal node *t* is split, and $\hat{I_{t}^{2}}$ is the reduction of the square loss (MSE) after the split of the internal node *t*. Therefore, the larger $\hat{I_{t}^{2}}$, the greater the ability of this node to reduce loss and the stronger the fitting ability.

The above feature importance score can discover which features have a greater impact on the final model, but it is impossible to discover the relationship between the features and the final prediction result. But the SHAP value can explore the relationship [28]. SHAP is an additive interpretation model inspired by Shapley Values. For each test sample, the model produces a predicted value, and the SHAP value is that assigned to each feature in the sample. Its calculation method is similar to the summation method of the linear model. By assuming that the model benchmark score (that is, the mean value of the target variable of all samples) is *y*_base_, the *i*^th^ sample is *x*_*i*_, the *j*^th^ feature of the *i*^th^ sample is *x*_*i*,*j*_, the SHAP value of this feature is *f*(*x*_*i*,*j*_), and the predicted value is

(3) $$\begin{array}{c} y_{i}=y_{\text{base}}+\;f\left(x_{i,1}\right)+f\left(x_{i,2}\right)+\cdots +f\left(x_{i,k}\right). \end{array}$$

When *f*(*x*_*i*,*j*_) > 0, the feature has a positive effect on the prediction of the target value and vice versa. Therefore, the SHAP value can not only reflect the influence of the overall characteristics but also the influence of the characteristics in each sample.

#### 3.1.4. Improvements to the Mean Processing of the Warning Period

**Figure 3:**
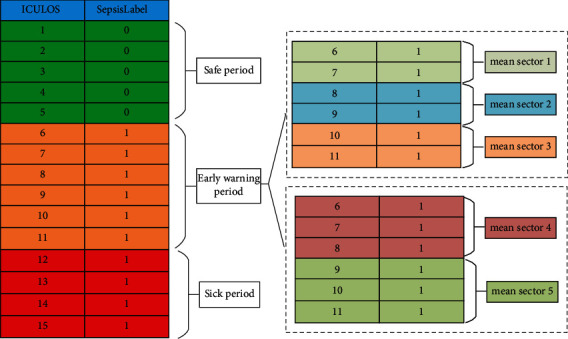
Diagram of dividing the 6 h early warning period into 2 h or 3 h time windows to calculate the mean vector.

In the above mean processing method, the 6-hour warning period for each patient is directly combined to obtain a single observation, but the prediction performance of the model may not be satisfactory. It is further explored whether when the time window of segmentation is finer or denser can lead to better performance or not. Therefore, the 6-hour warning period is divided into 2-hour or 3-hour time windows to calculate the mean vector, and the mean processing method of safe period and disease period is kept unchanged. The detail is shown in Figure 3. New datasets for training models are generated in the same way, and the improvement is compared with the original models of their generalization capabilities based on the ROC curve and P-R curve.

### 3.2. Feature Generation Method

Compared with the mean processing method, the feature generation method retains the original appearance of the data and valuable features are extracted as much as possible on the basis of the original features.

#### 3.2.1. Imputation of Missing Data

The original data set has more than 790,000 observations, with normal samples (patients without sepsis) accounting for 97.8%, and sepsis samples only accounting for 2.2%. As it differs a lot, the undersampling method is used to process the original data set. The method works by retaining the data with label 1 and undersamples the appropriate amount of data with label 0 to balance the category ratio.

When an individual is admitted into the ICU, the label may maintain as 0 in the early stage and transfer to stage 1 after a long time. Therefore, even for sepsis patients, the proportion of observations with label 1 does not exceed 20% on average. In this situation, only the data of sepsis patients are kept. The physiological data of all 1790 sepsis patients are selected from 22336 patients for analysis. The percentage of label category is shown in Table 3.

In the feature generation method, the forward deduction method is used for the original data, which means the latest value of a particular variable is used to estimate its missing value. If there are still missing values, the Miceforest imputation method is used to fill in the remaining missing values.

#### 3.2.2. Feature Generation

In order to explore more information from the original data in the feature generation method, this study considers generating new features from the original data by dividing time windows to extract statistical features [29]. Medical diagnostic indicators such as shock index and oxygenation index are also added as features to train the model.

The vital sign index data are generally measured more frequently and have a lower percentage of missing; however, the overall measurement frequency of laboratory test values is very low. In order to capture the physiological data detail, this study constructs two features: the total measurement counts of all vital signs and laboratory values till the current moment, and the corresponding inspection frequency. The specific calculation method is shown in Table 4.

According to the different frequencies of physiological data, time windows is constructed, and statistical features are extracted in each window. The time windows of different variables are shown in Table 5.

After the feature generation process, the variables included in the measurement intensity feature, window feature, and medical index feature are shown in Table 6.

The last three features are shown as follows. Shock index is expressed as pulse rate/systolic blood pressure (mmHg), namely, HR/SBP. The index can help measure the presence and severity of shock with a normal value of 0.5. When the shock index equals 1, it indicates mild shock; when the shock index is higher than 1.5, it indicates severe shock. One of the common symptoms of sepsis is the occurrence of shock, namely, insufficient tissue perfusion and continuous hypotension. Therefore, monitoring the shock index matters in predicting sepsis risk.

The oxygenation index is expressed as the percentage of arterial oxygen partial pressure/inspired oxygen concentration, namely, PaO_2_/FiO_2_. The normal value of this index is 400–500 mmHg. If PaO_2_ drops significantly, adding the oxygen concentration in the inhaled gas will not help to further increase PaO_2_. And if the oxygenation index is less than 300 mmHg, it suggests lung respiratory dysfunction happens. The diagnostic criteria for sepsis include unexplained hypoxemia, and the oxygenation index is an important indicator to monitor the patient's cardiopulmonary function. Unfortunately, arterial oxygen partial pressure PaO_2_ is not collected in the measurement data, but there is blood oxygen saturation SaO_2_ for replacement.

**Figure 4:**
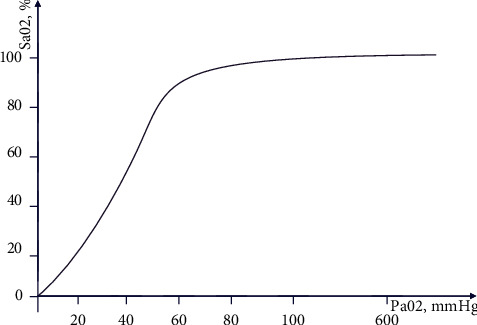
The oxygen dissociation curve.

It can be seen from the oxygen dissociation curve in Figure 4 that the level of oxygen saturation mainly depends on the level of oxygen partial pressure. The curve is S-shaped, and the change in the SaO_2_ value is generally positively correlated with the change in the PaO_2_ value. Therefore, SaO_2_/FiO_2_ is used to measure the patient's lung breathing function.

qSOFA score is mainly developed for sepsis screening in a resources-limited setting, as it does not require much intensive monitoring. As a sequential organ failure score, it measures whether the respiratory rate, systolic blood pressure, and state of consciousness are normal. If respiratory frequency ≥22 breaths/min, systolic blood pressure ≤100 mmHg, and change of consciousness happens in the qSOFA score table, one point will be added to the cumulative score. When the qSOFA score is greater than 2, the risk of sepsis becomes higher.

The number of new feature variables generated above and the original variables is 149 in total. Before training the model on these variables, some variables are eliminated. Among them, the entire column of EtCO_2_ was empty, so it is removed. The ICULOS variable and count variable are removed, because this study does not consider the relationship between the length of time in the ICU and whether patients suffer from sepsis. Finally, the number of variables entering the model training is 146. In the feature generation method, the same algorithms XGBoost and LightGBM are also applied along with the feature importance score and SHAP value.

## 4. Results

In the mean processing method and the feature generation method, 75% of the data are selected for training, and the remaining 25% are used in the test set for verification and evaluation. The result is shown in Table 7.

### 4.1. Model Performance

In the mean processing method (method1), the XGBoost and LightGBM algorithms differ in performance. The XGBoost algorithm has a recall rate of 0.55, with better distinction performance between 0-1 categories. From the comparison result between the Kappa coefficient and Matthews coefficient, it can be seen that the confusion matrix generated by the XGBoost algorithm is much more balanced in the test result.

**Figure 5:**
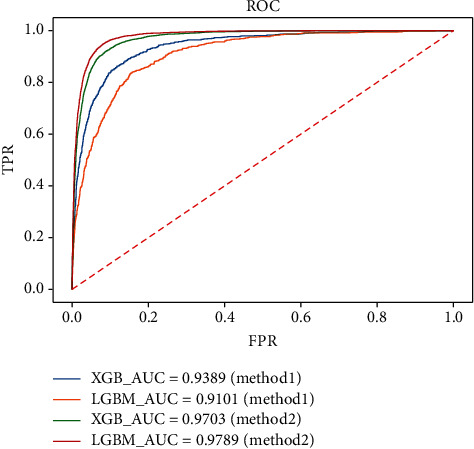
Comparison of AUC between different methods in XGBoost and LightGBM.

In the feature generation method (method2), the XGBoost and LightGBM algorithms perform better than the mean processing method. In the prediction result, the LightGBM model is better in precision, recall, and others with a better performance in distinguishing 0-1 categories. From the result of the Kappa coefficient and Matthews coefficient, it can be seen that the confusion matrix predicted by the LightGBM algorithm on the test set is more balanced, indicating that LightGBM is more excellent as shown in Figure 5. Overall, the feature generation method with LightBGM has the best performance.

### 4.2. Results of Improved Mean Processing Method

**Figure 6:**
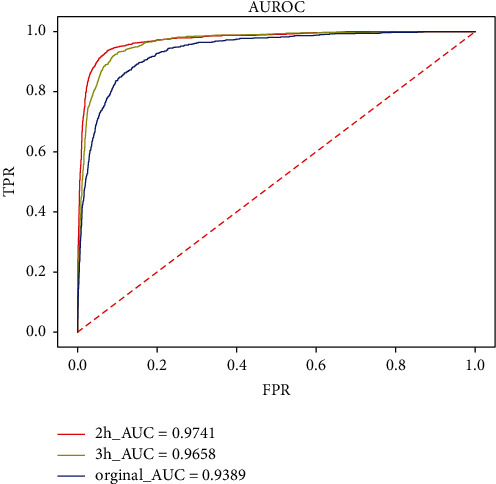
Comparison of AUC between the 2 h and 3 h improved model and the original model.

**Figure 7:**
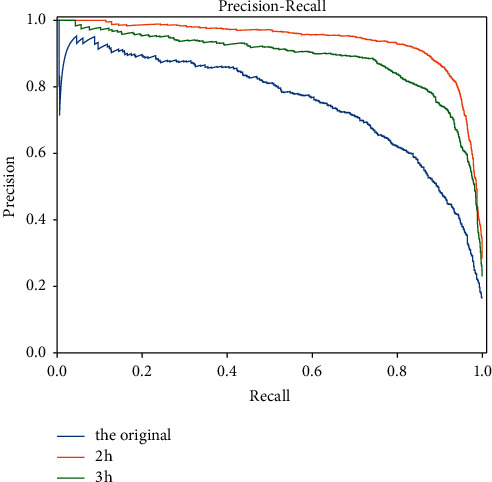
Comparison of P-R between the 2 h and 3 h improved model and the original model.

After dividing the time window into different sizes, the AUC value of the model trained on the data generated by the smallest 2 h time window significantly improves to 0.974. It also has the best performance in the P-R curve, which is about 4% higher than the accuracy of the original model. Figures 6 and 7 show that the more detailed the window feature extraction of time series data is, the more balanced the 0-1 distribution of categorical variables will be, so is the prediction performance of the model.

### 4.3. The Influence of the Features on the Model Performance

In the feature generation method, after adding measurement intensity features, window features, and medical indicator features, the prediction performance of the model further improves compared to the model trained directly with raw data. In order to explore the impact of the new features on the model prediction, the LightGBM algorithm is explored as an example. Respectively by deleting the features in the feature generation method and comparing the prediction effect of models containing different features on the test set, AUROC, Accuracy, and performance metrics like precision and recall are shown in Table 8.

From Table 8, it can be found that models trained with raw variables plus the measurement intensity feature or window feature or medical index feature are all helpful to improve the performance of the original model at various levels. Specifically, window features perform better than measurement intensity features and medical indicator features.

### 4.4. The Feature Importance

**Figure 8:**
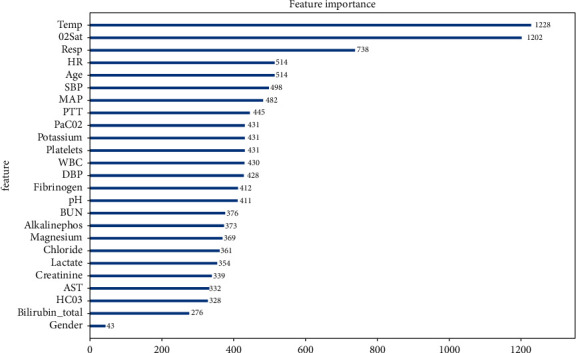
XGBoost algorithm's feature importance.

For the feature importance score, we take the XGBoost algorithm in the mean processing method as an example; the top 10 variables with feature importance scores are Temp, O2Sat, Resp, HR, Age, SBP, MAP, PTT, PaCO_2_, and Potassium as shown in Figure 8. This means that these variables play an important role in predicting the risk of sepsis.

The above feature importance score can show which features have a greater impact on the final model, but it is impossible to explore the relationship between the features and the final prediction result. The SHAP value can explore it.

**Figure 9:**
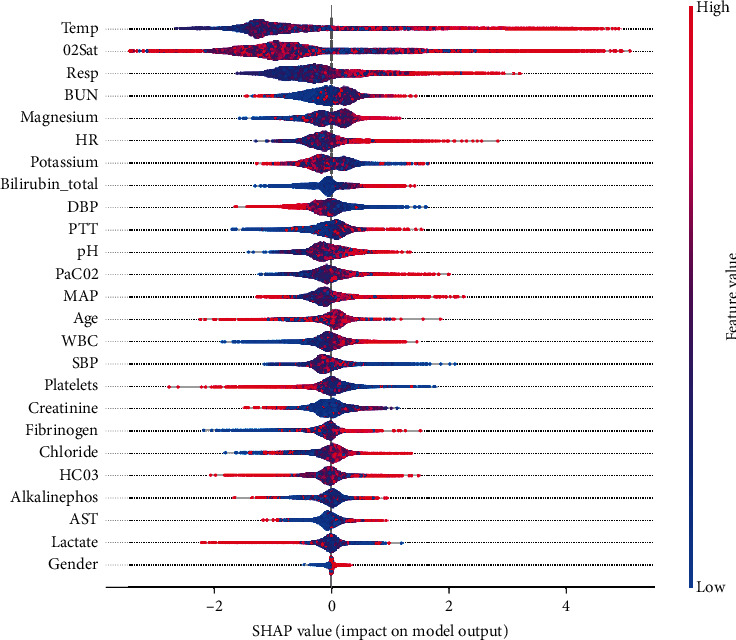
Sorted SHAP value diagram of all the features in the model.

As shown in Figure 9, each row in the figure represents a feature, the abscissa is the SHAP value, and a point represents a sample. The redder the color, the higher the SHAP value of the feature; the bluer the color, the lower the value of the feature. The wider area indicates a large number of features, where the sample is gathered. The ranking of the features is in descending order according to the average absolute value of SHAP. It is intuitively seen that Temp (temperature) is the most important feature for predicting early sepsis. The higher the value, the higher the risk of sepsis. Temp is followed by O_2_Sat (oxygen saturation), Resp (respiratory rate), BUN (blood urea nitrogen), and so on. The feature ranking of SHAP value is slightly different from the feature ranking of feature importance due to the difference in their calculation methods.

**Figure 10:**
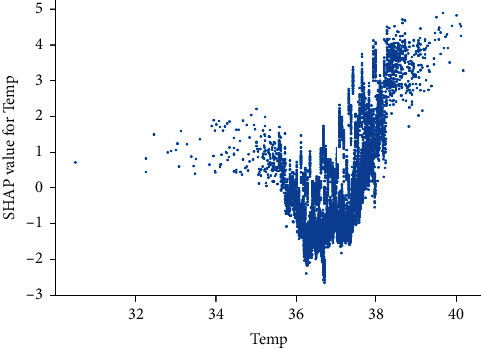
SHAP scatter plot of feature Temp.

In order to understand the corresponding relationship between the value of a single feature and the SHAP value, a scatter plot describing the corresponding relationship is suggested. The abscissa is the feature value, and the ordinate is the SHAP value corresponding to the characteristic value of all samples. As shown in Figure 10, taking the Temp as an example, when the temperature is lower than 36 degrees or higher than 37.8 degrees, the SHAP value will increase significantly, and the risk of early sepsis is greatly increased.

**Figure 11:**
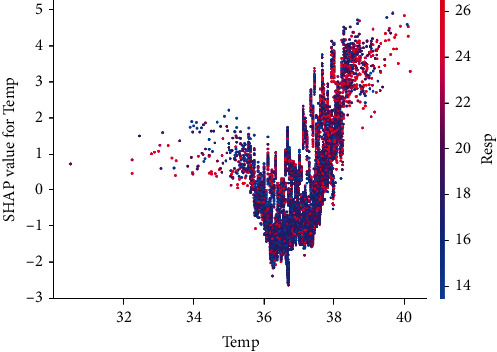
SHAP interaction scatter plot of feature Temp and Resp.

This interaction effect is further explored by adding a dependency, for example, the respiratory rate, as shown in Figure 11. Among them, in the upper half of the figure, the proportion of red points in all points is higher. This shows that for cases with higher Resp (respiratory rate), the temperature has a greater influence on predicting early sepsis.

The advantages of SHAP are that it can be displayed in each specific sample instance and reflect the important features which are affecting the results of prediction. The importance of these features, provided by the SHAP value, can be displayed by the length of the bar. The longer bar shape means that the SHAP value is higher and the variable is of greater importance. Among them, the feature that pushes the forecast higher (risk factor) is shown in red, and the feature that pushes the forecast lower (protection factor) is shown in blue.

**Figure 12:**
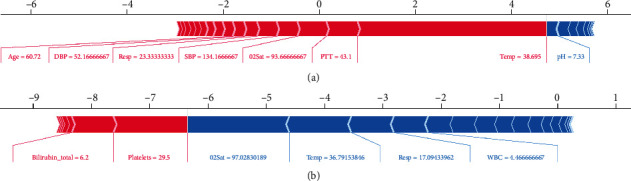
(a) Example of high risk according to SHAP value. (b) Example of low-risk according to SHAP value.

For example, Figure 12(a) shows a patient who keeps having a high temperature (>38.5 degrees Celsius), long thromboplastin time (PTT > 21), and low oxygen saturation. The model estimates that the sepsis risk of this patient is above average. In Figure 12(b), the patient is predicted as low sepsis risk. Although this patient has two risk factors: low platelet count (Platelets < 100) and high total bilirubin (Bilirubin_total > 1), its oxygen saturation, temperature, respiratory rate, and white blood cell count are all within the normal range, making the predicted risk of this case lower than the average.

## 5. Discussion and Conclusion

This study proposes two data processing methods and finds that the performance of the feature generation method trained with the LightGBM algorithm is the best, which can effectively explain the prediction results of each patient through SHAP value. For the mean processing method and the feature generation method, the performance highlights include the following. (1) The mean processing method streamlines the complicated data and avoids imputing a large amount of missing data, but the cost is that only the mean information is extracted from the data in different states, and the rest of the valuable information is lost, which limited the model's predictive ability. The improved mean processing method generates a new data set by calculating the mean vector of the divided warning period data per 2 hours and 3 hours, and the AUC of the model jumps to 0.97. (2) In the feature generation method, the amount of data obtained by undersampling contains about 100,000 observations. After filling in the missing values through Miceforest, the AUC of the original model reaches 0.971. Followed by generating new features, the improved model's AUC reached 0.979 and recall has the most obvious improvement from 0.6 to 0.64.

XGBoost has a stronger generalization ability when the amount of data and the number of features is relatively small. However, when the amount of data and the number of features increase sharply to a large scale, LightGBM has not only a fast iteration speed in training but also a better predictive ability than XGBoost. This is because it occupies low memory and adopts a leaf-wise growth strategy. It can be seen that when there are many types of data features and the scale of data is large, the LightGBM algorithm has obvious advantages in operating efficiency and memory.

PTT, WBC, and Platelets are important variables ranking top 10 in both mean processing method and feature generation method. These three laboratory values may be helpful to improve the accuracy of predicting early sepsis. Among them, WBC is an important indicator to determine whether inflammatory infection occurs. Increased white blood cell count is one of the most specific changes in acute bacterial infections. PTT and Platelets are coagulation indicators to assess organ function. Coagulation abnormalities are universal in patients with sepsis, which may cause multiple organ dysfunction. This will provide some evidence and hints for clinical diagnostic research.

The Miceforest algorithm has done excellently in filling in missing values. It can predict reasonable values for filling based on the distribution of the original data. Increasing the proportion of data of positive samples is a key to improve the performance of the model, which further fixes the problem of data imbalance while increasing the feature dimension to dig out more features is of limited help to improve the performance of the model.

The future work includes that further verification by prospective research is needed, given the unknown universality and stability of the model. More features of some variables can be mined to further explore variable information for better prediction performance. For imbalanced data, more effective methods can be studied to improve the generalization of the model.

## Figures and Tables

**Table 1:** Examples of variables in different types and the corresponding percentage of missing values.

Vital signs	Unit	Missing percentage
HR (heart rate)	Beats per minute	7.7%
O2Sat (pulse oximetry)	%	12.0%
Temp (temperature)	Deg C	66.2%
SBP (systolic BP)	Mm·Hg	15.2%
…	…	…

Laboratory variables	Unit	Missing percentage
BaseExcess (measure of excess bicarbonate)	mmol/L	89.6%
HCO3 (bicarbonate)	mmol/L	91.9%
FiO2 (fraction of inspired oxygen) pH	%	85.8%
pH	/	88.5%
…	…	…

Demographics	Unit	Missing percentage
Age	years	0.0%
Gender	Female (0) or male (1)	0.0%
Unit1 (MICU)	No(0) or yes(1)	48.9%
…	…	…

**Table 2:** The basic information of patients in ICU.

Basic information	Counts	Sum of counts	Proportion (%)	Sum of proportion (%)
Patients with sepsis	20662	22336	92.33	100
Patients with no sepsis	1714		7.67	
Sepsis label (0)	773080	790215	97.83	100
Sepsis label (1)	17135		2.17	

**Table 3:** The percentage of label category of undersampling.

Label	Counts	Ratio of labels 1 and 0
0	88828	5.18: 1
1	17136	
Sum	105964	

**Table 4:** Measurement intensity calculation method.

Features	Calculation method
Total count	The total count of vital signs indicators and laboratory values from ICULOS = 1 to ICULOS = *t* (*t* is the current time)
Inspection frequency	Count(*t*)/ICULOS(*t*)

**Table 5:** Time window structure of different variables.

Variables	Time window length	Feature descriptions
HR, MAP, O2Sat, Resp, SBP	2 hours	Calculate the difference between values when ICULOS = *t* or ICULOS = t-2
WBC, Temp, MAP, SBP, Creatinine, Platelets, FiO2, SaO2, PTT, BUN, Calcium, Phosphate, Hct, Lactate, Alkalinephos, Glucose, Hgb	12 hours	Calculate maximum, minimum, and the time range from ICULOS = t-12 and t-24 to ICULOS = *t*
	24 hours	

Note: (1) when the length of the time window is 2 h, if the current time *t* < 2, the feature value is nan, and the missing position is filled with the median of the column data; (2) when the length of time windows is 12 h/24 h, if the current time *t* < 12/24, the time window will change into time from ICULOS = 1 to the current time ICULOS = *t* to construct features.

**Table 6:** The meaning of the new feature variable.

Features	Based variables	Descriptions
Count	All vital signs data + all laboratory value data	The count of the measurement times of all variables till the current moment
Inspection frequency		The sum of the measurement times of all variables per unit time till the current moment
2h_diff	Some vital signs variables^1^	The difference between the current time and the value 2 hours ago
12h_max	Some laboratory value variables^2^	Maximum value in a sliding time window spanning 12 hours
12h_min		Minimum value in a sliding time window spanning 12 hours
12h_maxmin		Range in a sliding time window spanning 12 hours
24h_max		Maximum value in a sliding time window spanning 24 hours
24h_min		Minimum value in a sliding time window spanning 24 hours
24h_maxmin		Range value in a sliding time window spanning 24 hours
HR/SBP	HR,SBP	Shock index
SaO_2_/FiO_2_	SaO_2_, FiO_2_	Oxygenation index
qSOFA	Resp, SBP	qSOFA score

^1^HR, MAP, O2Sat, Resp, and SBP. ^2^WBC, Temp, MAP, SBP, Creatinine, Platelets, FiO_2_, SaO_2_, PTT, BUN, Calcium, Phosphate, Hct, Lactate, AlkalinePhos, Glucose, and Hgb.

**Table 7:** Prediction ability of different methods in XGBoost and LightGBM.

		Precision	Recall	F1-score	Kappa coefficient	Matthews coefficient
Mean processing method	XGBoost	0.78^*∗*^	0.55^*∗*^	0.65^*∗*^	0.60^*∗*^	0.61^*∗*^
	LightGBM	0.70	0.42	0.53	0.46	0.48

Feature generation method	XGBoost	0.89	0.61	0.72	0.67	0.69
	LightGBM	0.91^*∗*^	0.65^*∗*^	0.76^*∗*^	0.72^*∗*^	0.73^*∗*^

**Table 8:** Prediction performance of different features on the test set.

Variables	AUROC	Accuracy	Precision	Recall
Raw variables	0.9710	0.919	0.92	0.60
Raw variables + measurement intensity features	0.9756	0.927	0.93	0.64
Raw variables + window features	0.9767	0.928	0.93	0.64
Raw variables + medical indicator features	0.9718	0.919	0.92	0.60
Feature generation method features	0.9789	0.931	0.93	0.65

## Data Availability

The data set used for this study is openly available and the details are mentioned in the article. The data sets used in this study can be found on the website https://archive.physionet.org/users/shared/challenge-2019/.
